# Involvement of Fas/FasL pathway in the murine model of atopic dermatitis

**DOI:** 10.1007/s00011-017-1049-z

**Published:** 2017-04-22

**Authors:** Karolina Bień, Magdalena Żmigrodzka, Piotr Orłowski, Aleksandra Fruba, Łukasz Szymański, Wanda Stankiewicz, Zuzanna Nowak, Tadeusz Malewski, Małgorzata Krzyżowska

**Affiliations:** 1 Department of Regenerative Medicine, Military Institute of Hygiene and Epidemiology, Kozielska 4, 01-163 Warsaw, Poland; 2 Department of Pathology and Veterinary Diagnostics, Faculty of Veterinary Medicine, Warsaw University of Life Sciences, Nowoursynowska 159c, 02-776 Warsaw, Poland; 3 Department of Genetics and Animal Breeding, Faculty of Animal Science, Warsaw University of Life Sciences, Ciszewskiego 8, 02-786 Warsaw, Poland; 4 Museum and Institute of Zoology, Polish Academy of Science, Wilcza 64, 00-679 Warsaw, Poland

**Keywords:** Apoptosis, Fas/FasL, Atopic dermatitis, Ovalbumin, Inflammation

## Abstract

**Objective and design:**

The aim of this study was to elucidate the role of apoptosis mediated through Fas/FasL pathway using the mouse model of atopic dermatitis (AD).

**Materials and treatment:**

AD was induced by epicutaneous application of ovalbumin (OVA) in wild-type C57BL/6, B6. MRL-Faslpr/J (Fas−) and B6Smn.C3-Faslgld/J (FasL−) mouse strains.

**Methods:**

Skin samples were subjected to staining for Fas/FasL expression, M30 epitope and assessment of inflammatory response via immunohistochemical staining. Cytokine and chemokine production was assessed by real-time PCR.

**Results:**

In comparison to wild-type mice, OVA sensitization of Fas- and FasL-deficient mice led to increased epidermal and dermal thickness, collagen deposition and local inflammation consisting of macrophages, neutrophils and CD4+ T cells. Fas- and FasL-deficient mice showed increased total counts of regulatory T cells (Tregs) and IgE levels in blood as well as increased expression of IL-1β, IL-4, IL-5, IL-13 and TGF-1β mRNA in comparison to wild-type mice. On the other hand, expression of CXCL9 and CXCL10, IL-17 mRNAs in the skin samples in Fas- and FasL-deficient mice was decreased.

**Conclusions:**

Our results show that lack of the Fas-induced apoptosis leads to exacerbation of AD characteristics such as Th2 inflammation and dermal thickening. Therefore, Fas receptor can play an important role in AD pathogenesis by controlling development of the local inflammation.

## Introduction

Atopic dermatitis (AD) is a common allergic skin disorder characterized by chronic relapsing form of skin inflammation, disturbance of epidermal barrier function that leads to dry skin, and keratinocyte apoptosis as a mechanism of eczema and spongiosis formation, which is mostly seen in acute and subacute lesions. The pathogenesis of AD is multifactorial including genetic, environmental, skin barrier, psychological and immunological factors [1, 2]. During AD, the local response of keratinocytes together with the reaction of endothelial cells, T cells, mast cells, macrophages, eosinophils and dendritic cells finally leads to the characteristic clinical and histological appearance of AD [1, 2].

Several studies have demonstrated that keratinocyte apoptosis is an important component of AD and is mediated through Fas/FasL pathway [3, 4]. Apoptosis is an active, genetically controlled process, which can be triggered by a variety of extrinsic and intrinsic signals [5]. Apoptosis in the skin represents a key event of the epidermal homeostasis: apoptosis removes exceeding cells and guarantees the normal epidermal architecture, it also represents an important cancer defence mechanism in response to UV radiation or oxidative damage [6, 7].

Two major signalling apoptotic pathways have been discovered: the receptor-ligand-mediated pathway and the mitochondrial-driven pathway. Fas (CD95) and other receptors from the tumour necrosis factor (TNF) family upon interaction with their ligands, (e.g. FasL) trigger the so-called death-receptor pathway of apoptosis. Epidermal keratinocytes express Fas in low amounts [8]. In inflammatory and infectious dermatoses, IFN-γ increases Fas expression [3, 4]. FasL is absent in normal skin, but is constitutively expressed on histiocytes in the dermis [9]. Abnormal expression of lytically active FasL was found in inflammatory skin diseases such as toxic epidermal necrolysis, atopic dermatitis and allergic contact dermatitis [10]. After stimulation with pro-inflammatory cytokines such as IL-1 β, TNF-α, IFN-γ and IL-15, but not IL-10, IL-12, TGF-β, keratinocytes express in a time- and dose-dependent manner FasL [3, 4].

Signalling via death receptors plays a distinct role in the immune system, where it contributes to regulation of the adaptive immune response in various ways, most notably by triggering apoptosis of T cells [11] and also by the elimination of inflammatory cells [12].

Emerging evidence indicates that Fas/FasL death receptors activate inflammatory or proliferative signalling via the prototypic pro-inflammatory transcription factor NF-κB or the mitogen-activated protein kinase (MAPK) family of kinases [13]. Farley et al. [14, 15] demonstrated that FasL triggered an NF-kB-dependent mRNA accumulation of inflammatory cytokines (TNF-α, IL-6 and IL-1β), chemokines (CCL2, CXCL1, CXCL3 and CXCL8/IL-8), and the adhesion molecule ICAM-1 in HaCaT cells and in reconstructed human epidermis (RHE). Activation of Fas was required both for apoptosis and for gene expression. Also Krzyzowska et al. [12, 16] showed that murine keratinocytes stimulated with Fas cytotoxic antibody start to produce TNF-α, CXCL10 and IL-1β.

Therefore, death receptors, such as Fas/FasL, may induce non-apoptotic signals and shape the quality and quantity of the cytokine and chemokine cocktail produced during the effector phase of AD. These functions may have important and distinct pathophysiological consequences during different stages of AD inflammatory reaction.

Here, we applied the model developed by Spergel et al. [17] to wild-type C57BL/6, B6. MRL-Faslpr/J (Fas−) and B6.Smn.C3-Faslgld/J (FasL−) mouse strains to study how Fas/FasL receptors influence development of AD induced by epicutaneous application of sensitizer—ovalbumin.

## Methods

### Animals and experimental procedure

Mice of both sexes, 5- to 7-week old, were used for all the experiments. B6. MRL-Faslpr/J (Fas−) and B6.Smn.C3-Faslgld/J (FasL−) mice were purchased from the Jackson Laboratory (Bar Harbor, ME, USA) and a breeding colony was maintained at the Oncology Centre (Warsaw, Poland) animal facilities. C57BL/6 mice were purchased from the Mossakowski Medical Research Centre (Warsaw, Poland) and used as wild-type controls. This study was performed in accordance with the recommendations of the Polish Act of 21 January 2005 on animal experiments (OJ no 33, item 289) and Directive 2010/63/EU of the European Parliament and the Council of 22 September 2010 on the protection of animals used for scientific purposes. The protocol was approved by the 4th Local Committee on the Ethics in Animal Experiments in Warsaw, Poland (Permit Number: 84/2012).

The mouse model of AD was performed according to the procedure described by Spergel et al. [17]. The mice were anesthetized and then shaved with a razor. One hundred µg of ovalbumin (OVA) in 100 µl of saline was placed on a patch of sterile gauze (1 × 1 cm), which was further secured to the skin with a transparent occlusive dressing. The patches were placed for a 1-week period and then removed. Two weeks later, an identical patch was applied to the same skin site. Each mouse had a total of three 1-week exposures to the patch separated from each other by 2-week intervals. All mice from three strains underwent the same procedure. At the end of the third sensitization, the following samples were obtained: blood and skin from the saline and OVA-stimulated area. The experiment was repeated three times with the groups of 7 mice per each strain.

### Collagen and skin thickness measurements

Skin samples were fixed in 4% paraformaldehyde in PBS for 24h, then dehydrated and embedded in paraffin. The 6-µm sections were further stained with Masson’s Trichrome to evaluate collagen, according to the producer’s protocol (Sigma-Aldrich, St. Louis, MO, USA). Epidermal and dermal thickening was quantitated using the Zeiss Axio Scope.A1 microscope and ZEN software (Zeiss, Oberkochen, Germany).

### Immunohistochemical staining

Skin samples were de-paraffinized by sequential placement in xylene and ethanol, then subjected to antigen retrieval in 0.1 Mcitrate buffer (pH 6.0) for 10 min. Fas and FasL were detected using monoclonal hamster anti-mouse Fas antibody (Jo-2) and monoclonal hamster anti-mouse FasL antibody (MFL3) (BD Biosciences, Franklin Lakes, NJ, USA). Briefly, after 30 min of incubation with primary antibody diluted in 1% bovine serum albumin/PBS at RT (1:100), secondary biotinylated anti-hamster IgG antibody (BD Biosciences) (1:250) in PBS, then streptavidin-HRP (1:300) (BD Biosciences) was added for 30 min. Immunofluorescence staining for Fas and FasL was carried out using FITC-conjugated hamster anti-mouse Fas antibody (Jo-2) and PE-conjugated hamster anti-mouse FasL antibody (MFL4) were used (BD Biosciences), as described above. After mounting the slides in medium containing Hoechst 33342 (1 µg/ml), fluorescence was captured with ArrayScan™ XTI High Content Platform equipped with HCS Studio™ 2.0 Cell Analysis Software (Thermo Fisher Scientific Inc., Waltham, MA, USA).

Apoptotic cells were detected with the M30 CytoDEATH antibody recognizing a specific caspase-cleavage site within cytokeratin 18 in apoptotic cells (1:100) (Sigma-Aldrich) according to the producer’s protocol. After 30 min of incubation with primary antibody diluted in 0.05 mol/L Tris–HCl buffer, pH 7.2, 1% bovine serum albumin at RT, PE-conjugated goat anti-mouse immunoglobulins were added for another 30 min (1:250). After mounting the slides in the medium containing Hoechst 33342 (1 µg/ml), fluorescence was captured with Leica TCS SP5 II confocal microscope (Leica, Wetzlar, Germany).

### Immunophenotyping

De-paraffinized skin samples were subjected to antigen retrieval, as described above. After blocking and incubation in PBS containing 2% FBS for 30 min, samples were stained with rat anti-CD11b FITC (M1/70), rat anti-Ly6-G/Ly6-G-PE (Gr-1, clone RB6-8C5), rat anti-CD3e-FITC (145-2C11), rat anti-CD4-PE (RM4-5), rat anti-CD8-PE (53-6.7.) and rat anti-F4/80 APC (e-Biosciences, San Diego, CA, USA) (1:100) for 1h in room temperature. After mounting the slides in medium containing Hoechst 33342 (1 µg/ml), fluorescence was captured with ArrayScan™ XTI High Content Platform equipped with HCS Studio™ 2.0 Cell Analysis Software (Thermo Fisher). To define the positive objects, intensity threshold for double staining was used as object finding module. Selected parameters such as counts, were estimated for each fluorescence channel. The values are presented as the number of double-positive events per the same area of each skin sample.

### Cell characterization by flow cytometry

Leucocytes from blood were isolated utilizing Histopaque 1119 (Sigma–Aldrich) by centrifugation 30 min × 600*g*. Cell suspensions were pretreated with the Fc receptors block-rat anti-CD16/32 antibody (2.4G2) (BD Biosciences) according to the manufacturer’s protocol. Tregs were detected using BD Pharmingen™ Mouse Foxp3 Buffer Set, anti-CD4-PE (see above), anti-CD25-FITC (7D4) and rat anti-Foxp3+-Alexa 657 (MF23) antibodies (BD Biosciences). For all phenotyping, rat IgG2a, rat IgG2b and hamster IgG1 isotype antibodies conjugated with appropriate fluorochromes were used (BD Biosciences). The stained cell suspensions were analysed in FACS Calibur for positively stained cells. The total cell count was set at 100.000 events per each experiment.

### Quantitative reverse transcriptase-polymerase chain reaction (RT2-PCR)

Total DNA was isolated from the liver and spleen tissues preserved in RNA later (Sigma-Aldrich) using Universal DNA/RNA/Protein Purification Kit (Eurx, Gdansk, Poland). For cytokine and chemokine quantification, cDNA was reverse-transcribed from 1 μg of total RNA using SuperScript® III One-Step RT-PCR System with Platinum® Taq DNA Polymerase (Thermo Fisher Scientific) according to the manufacturer’s protocol. To analyse expression of selected cytokines and chemokines we used the following primers: *IL-4*: 5′CGGAGATGGATGTGCCAAAA3′ and 5′GCACCTTGGAAGCCCTACAG3′; *IL-5*: 5′CTCTGTTGACAAGCAATGAGACG3′ and 5′TCTTCAGTATGTCTAGCCCCTG3′; *IL-13*: 5′CAGCATGGTATGGAGTGTGG3′ and 5′TGGGCTACTTCGATTTTGGT3′; 5′CATGGTCCTGAGACAAAAGT3′ and 5′CATGGTCCTGAGACAAAAGT3′. *Fas*: 5′CAGACATGCTGTGGATCTGG3′ and 5′CACAGTGTTCACAGCCAGGA3′; *FasL*: 5′CAGCTCTTCCACCTGCAGAAG3′ and 5′AGATTCCTCAAAATTGATCAGAGAGAG3′; *Gapdh* was used as a reporter gene in our experiment: 5′GCCACATTCTATACAGGGATTGG3′ and 5′GCCACATTCTATACAGGGATTGG3′. Reactions with the starters above were carried out using LuminoCt SYBR Green qPCR Master Mix (Sigma-Aldrich, St. Louis, MO, USA) in the RotorGene 6000 system. Transcripts of IL-1β, IL-10, IL-17, TGF-β1, CXCL9 and GADPH were quantified using Taqman(R) Gene Expression Assays with TaqMan Gene Expression Master Mix (Applied Biosystems, Foster City, CA, USA) using 7500 Real-Time PCR System (Applied Biosystems) according to the manufacturer’s protocol. The ${{\text{2}}^{\Delta \Delta {C_{\text{t}}}}}$ method was used in calculating the relative ratio, but instead of value 2, the correct amplification efficiency was used [18]. mRNA levels were counted from three PCR reactions for each sample.

### IgE quantification

The IgE antibodies levels in sera obtained from OVA-treated and untreated mice after third round of sensitization were tested using Mouse IgE Ready-SET-Go! Kit (e-Biosciences) according to the manufacturer’s protocol.

### Statistical methods

The obtained data were subjected to the W. Shapiro–Wilk test for normality and the Levene’s test for equality of variances. For normal distribution of values, statistical comparisons were performed using the Student’s *t* test. For data following non-Gaussian distributions, non-parametric Wilcoxon for dependent samples and Kruskal–Wallis test with post hoc multiple comparisons for comparison of all pairs were applied. Quantitative data were presented as means ± SEM. In every analysis, values of *p* ≤ 0.05 were considered significant. All calculations were performed with Statistica 8.0 (Statsoft).

## Results

### Epicutaneous sensitization with OVA induces Fas and FasL expression

**Fig. 1:**
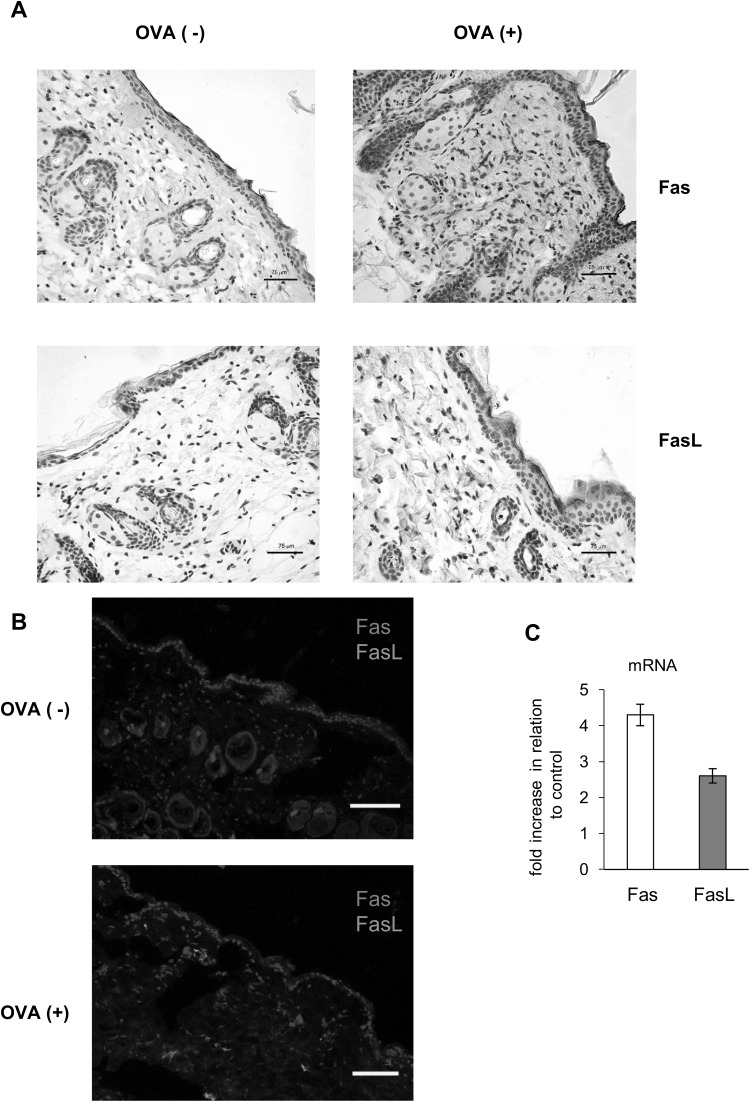
Epicutaneous sensitization with ovalbumin (OVA) induces Fas and FasL expression. **a** Representative images of Fas and FasL expression identified by immunohistochemistry method in the in paraffin-embedded slides prepared from the skin of C57BL/6 mice, sensitized with saline (OVA−) or ovalbumin (OVA+). The nuclei were counterstained with Harris hematoxylin (*violet*). Magnification ×200. **b** Double staining for Fas (*red*) and FasL (*green*) by immunohistofluorescence method in the in paraffin-embedded slides prepared from the skin of C57BL/6 mice, sensitized with saline (OVA−) or ovalbumin (OVA+). The nuclei were counterstained with Hoechst 33342 (*blue*). *White scale bar* shows 75 µm. **c** Relative mRNA levels of Fas and FasL in the skin of C57BL/6 mice, sensitized with ovalbumin (OVA+). The mRNA expressions were normalized by that of GAPDH, and showed as fold increase in relation to saline-sensitized skin samples—control OVA (−). The* bars* represent the mean from 3 separate experiments ± SEM (color figure online)

Since epidermal keratinocytes express Fas in low amounts [8] and FasL expression is detected in the inflammatory skin conditions [10], first we accessed Fas and FasL expression in the skin of mice subjected to ovalbumin (OVA) or saline epicutaneous sensitization (EC) (Fig. 1a).

Wild-type C57BL6/j mice not subjected to sensitization with OVA showed weak Fas expression only on single cells of leucocyte morphology present in the dermis and no detectable FasL expression (Fig. 1a, b). Epicutaneous sensitization with OVA of wild-type mice led not only to up-regulation of Fas expression on the cells infiltrating the dermis and epidermis but also on the keratinocytes localized at stratum spinosum (Fig. 1a, b). FasL expression was detected only on the cells infiltrating the dermis and epidermis (Fig. 1a, b).

RT2-PCR quantification of mRNA for Fas and FasL showed significant increase in Fas and FasL expression in mouse subjected to OVA epicutaneous sensitization when compared to saline sensitization (*p* ≤ 0.05) (Fig. 1c). Increase in Fas expression was higher than FasL expression (Fig. 1c).

### Epicutaneous sensitization with OVA of Fas- and FasL-deficient mice leads to increased local inflammation in the skin

**Fig. 2:**
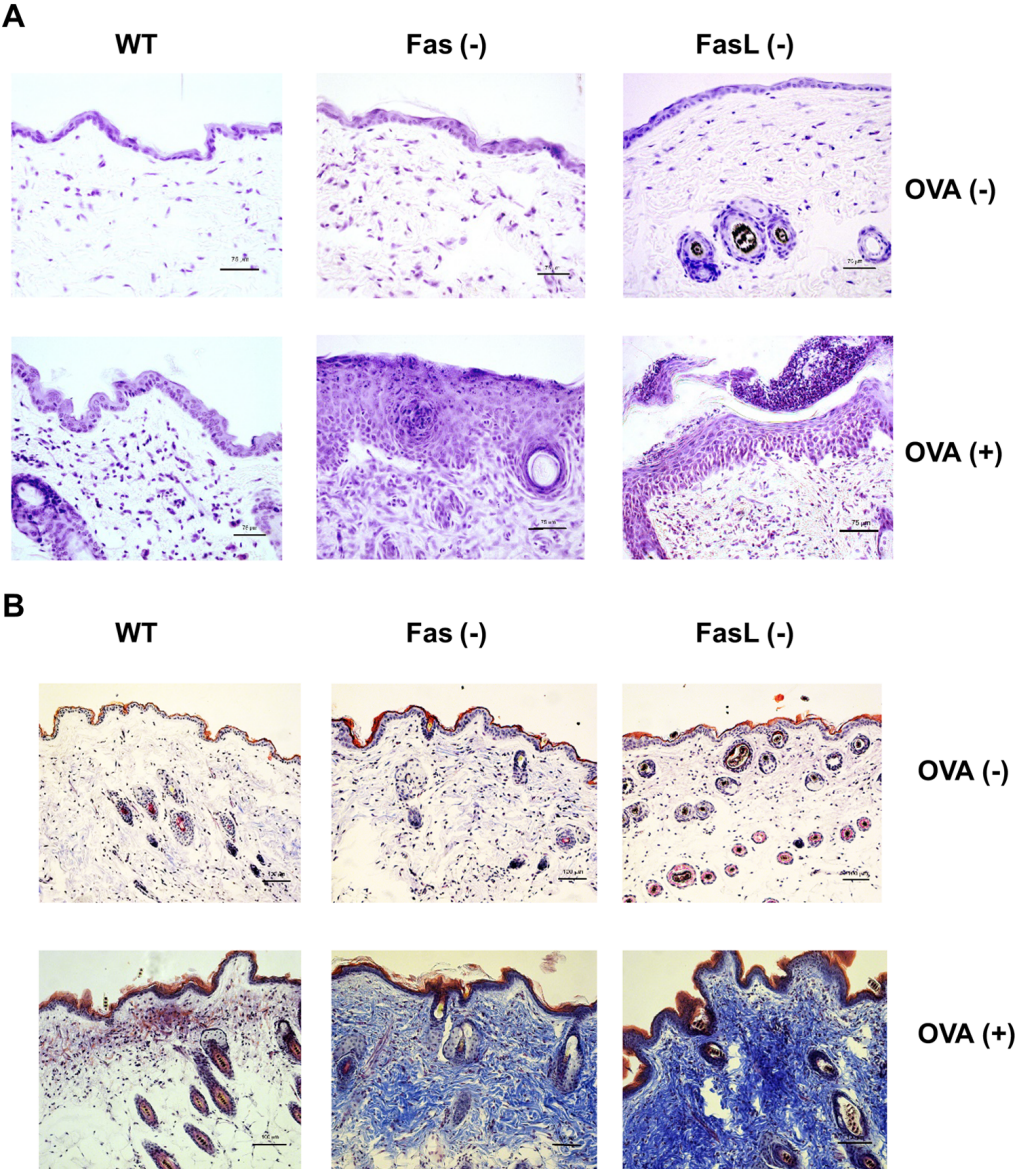
Histological features of OVA-sensitized and saline-sensitized skin samples from C57BL/6 (WT), B6. MRL-Faslpr/J (Fas−) and B6Smn.C3-Faslgld/J (FasL−) mice. **a** Skin sections were stained with Harris hematoxylin. **b** Masson’s trichrome staining for collagen deposition

**Fig. 3:**
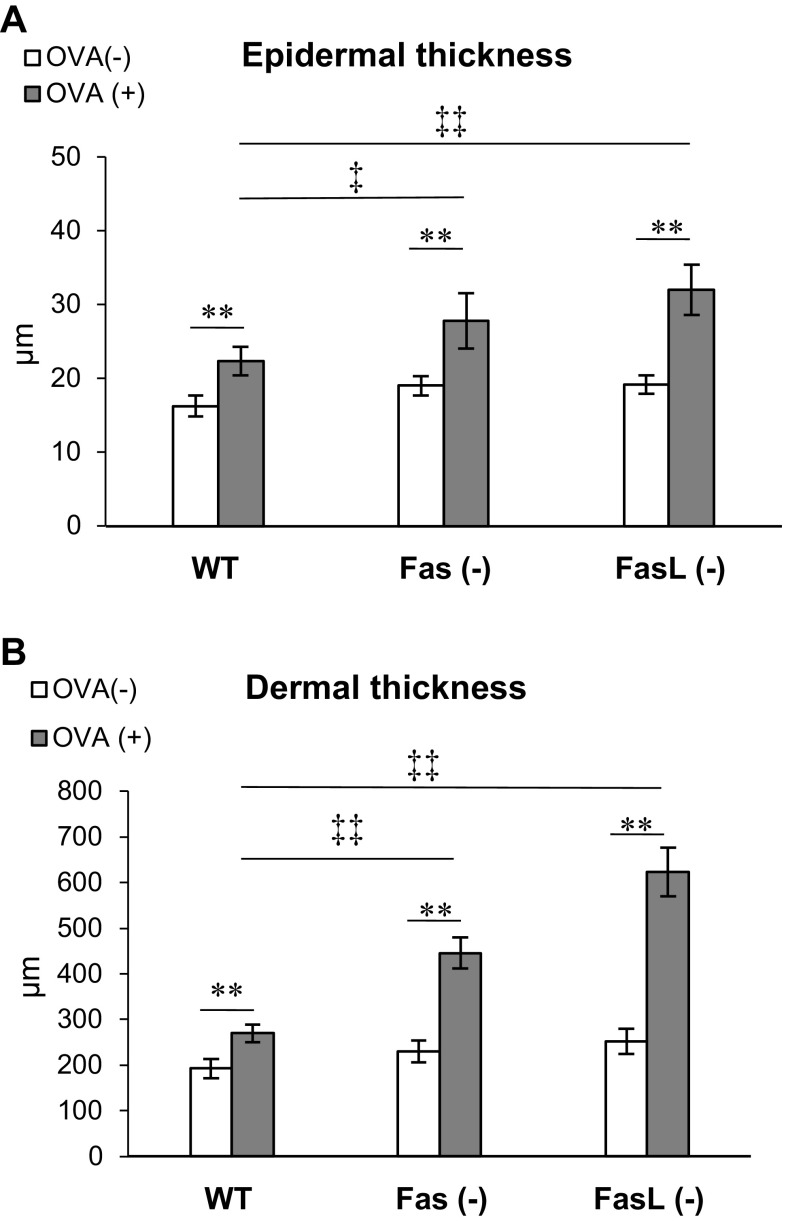
Quantification of epidermal and dermal thickening in OVA-sensitized and saline-sensitized skin samples from wild-type, Fas (−) and FasL (−) mice. Values are mean ± SEM from 10 mice. **Significant differences with *p* ≤ 0.001 in comparison to saline-treated control, while ^‡^significant differences with *p* ≤ 0.05, ^‡‡^ *p* ≤ 0.001 in comparison to wild-type OVA-sensitized controls

To elucidate the role of Fas/FasL pathway in the local inflammation induced by epicutaneous sensitization with OVA, we used wild-type C57BL6 mice and Fas- or FasL-deficient mice for the mouse model of AD. The skin from the OVA-sensitized wild-type mice showed thickening and inflammation in the dermis and epidermis at the site of epicutaneous sensitization with OVA, but not with saline (Fig. 2a). The epidermis of the OVA-sensitized skin sites exhibited the presence of the focal acanthosis, lymphocyte and neutrophil infiltration (Fig. 2a). The dermal layer of the OVA sensitized, but not of the saline-sensitized mice was infiltrated with neutrophils, lymphocytes, mast cells and eosinophils (Fig. 2a, b). We quantified the thickness of both layers in the epidermis and dermis together by examining 20 fields (Fig. 3a, b). Upon measurement, the epidermis and dermis in wild-type mice was thicker in the OVA-sensitized mice compared with the control, saline-sensitized mice (*p* ≤ 0.05) (Fig. 3a, b). Epicutaneous sensitization of Fas- and FasL-deficient mice led to significantly higher thickening and inflammation of both epidermal and dermal layer in comparison to wild-type mice (*p* ≤ 0.05) (Figs. 2, 3). The epidermis of OVA-sensitized Fas- and FasL-deficient mice was several layers thicker with stronger focal acanthosis, neutrophil infiltration and signs of spongiosis (Fig. 2a, b). The dermal layer of OVA-sensitized Fas- and FasL-deficient mice was characterized with strong infiltration of neutrophils, lymphocytes and eosinophils (Fig. 2a, b). Masson’s Trichrome staining revealed that OVA-sensitized Fas- and FasL-deficient mice had also shown the highest deposition of collagen fibres in papillary and reticular layers of the dermis (Fig. 2b). Moreover, OVA-treated Fas- and FasL-deficient mice also presented the highest number of fibroblasts in the reticular dermis, leading to significant skin fibrosis in comparison to wild-type mice (Fig. 2b).

To further characterize the cellular infiltrate in the skin, we performed immunochemistry analysis on the skin samples. The skin samples from OVA-sensitized Fas- and FasL-deficient mice showed significantly higher infiltration with macrophages (F4/80+/CD11b+) (*p* ≤ 0.01), neutrophils (*p* ≤ 0.01) (Gr-1+/CD11b-) and CD4+ T cells (*p* ≤ 0.05) (CD3+/CD4+) in comparison to skin samples from OVA-sensitized wild-type mice (Table 1). CD8+ T cells were identified as single cells without significant difference between tested mice strains (data not shown). No differences were observed between mice strains in the skin samples from saline-sensitized mice.

**Table 1:** Quantification of the cellular infiltrate of the OVA− and saline-sensitized skin sites in wild-type (WT), Fas (−) and FasL (−) mice

	WT	Fas (−)	FasL (−)
Macrophages			
OVA (−)	52.56 ± 7.4	51.03 ± 4.55	58.65 ± 10.11
OVA (+)	102.5 ± 11.83	154.05 ± 17.23**	164.83 ± 18.9**
Neutrophils			
OVA (−)	3.4 ± 0.56	4.5 ± 0.22	4.05 ± 0.6
OVA (+)	28.1 ± 4.5	68 ± 7.33**	75.34 ± 8.01**
CD4+ T cells			
OVA (−)	2.34 ± 0.98	3.81 ± 1.1	2.99 ± 0.79
OVA (+)	45.81 ± 6.2	61.5 ± 5.3*	68.2 ± 6.97*

The results are mean ± SEM from three experiments. *Significant differences with *p* ≤ 0.05, while ** means *p* ≤ 0.01

**Fig. 4:**
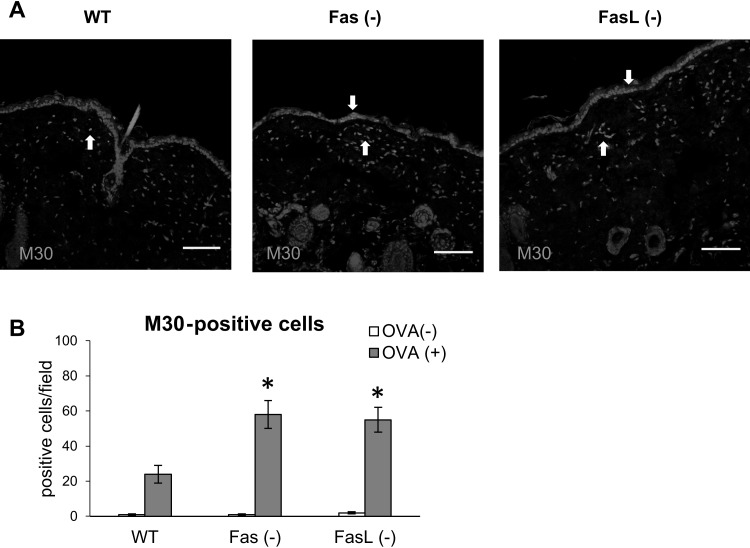
Quantification of apoptotic cells in OVA-sensitized and saline-sensitized skin samples from wild-type, Fas (−) and FasL (−) mice. **a** Representative images of M30 + cells (*red*) identified by immunofluorescent method in the in paraffin-embedded slides prepared from the skin of C57BL/6 mice Fas (−) and FasL (−) mice, sensitized with ovalbumin (OVA+). The nuclei were counterstained with Hoechst 33342 (*blue*). *White arrow* indicates positive cells. *White scale bar* shows 100 µm. **b** Total M30 + cells/field were enumerated in skin samples that included both dermis and epidermis. Values are mean ± SEM from 10 mice. *Significant differences with *p* ≤ 0.05 in comparison to wild-type mice

We also tested for the numbers of early apoptotic (M30+) cells in the skin samples from control and OVA-sensitized skin samples. In the wild-type mice, apoptotic cells were detected as single infiltrating cells within the dermis and single keratinocytes at the basement membrane (Fig. 4a). In Fas- or FasL-deficient mice, apoptotic cells were detected mostly as the cells infiltrating dermis or keratinocytes around the areas of inflammatory cells migration into the epidermis (Fig. 4a). Quantification of the numbers of apoptotic cells in the skin samples of OVA-sensitized wild-type, Fas- and FasL-deficient mice showed significantly higher numbers of M30 + cells (*p* ≤ 0.05) (Fig. 4b).

### Foxp3 + regulatory cells (Tregs) induction

**Fig. 5:**
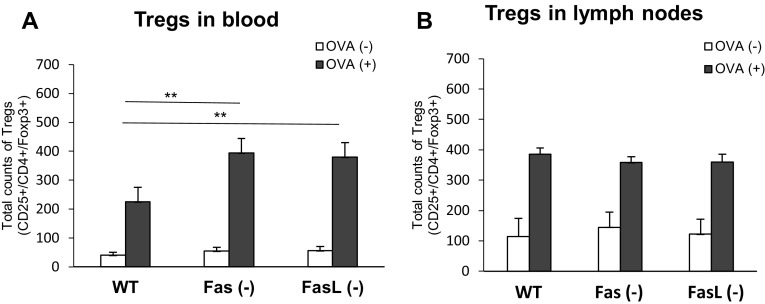
Total counts of Tregs (CD4+/CD25+/Foxp3+) in blood (**a**) and lymph nodes (**b**) from OVA-sensitized and saline-sensitized Fas (−), FasL (−) and WT (C57BL/6) mice. The* bars* represent the mean from 3 separate experiments ± SEM. **Significant differences with *p* ≤ 0.01 in comparison to wild-type mice

In this study, we have measured total counts of Foxp3+/CD25+/CD4+ T cells (Tregs) in blood from OVA- or saline-sensitized wild-type, Fas- and FasL-deficient mice. As demonstrated in Fig. 5a, a significant increase in the total counts of Tregs in blood was observed in OVA-sensitized Fas (−) and FasL (−) mice compared with the OVA-sensitized wild-type mice (*p* ≤ 0.01). No significant differences were observed in saline-sensitized wild-type, Fas- and FasL-deficient mice (Fig. 5). For the draining lymph nodes, we observed no significant differences in the total counts of Tregs (Fig. 5b) both in saline and OVA-sensitized mice.

### Fas/FasL pathway influences IgE production

**Fig. 6:**
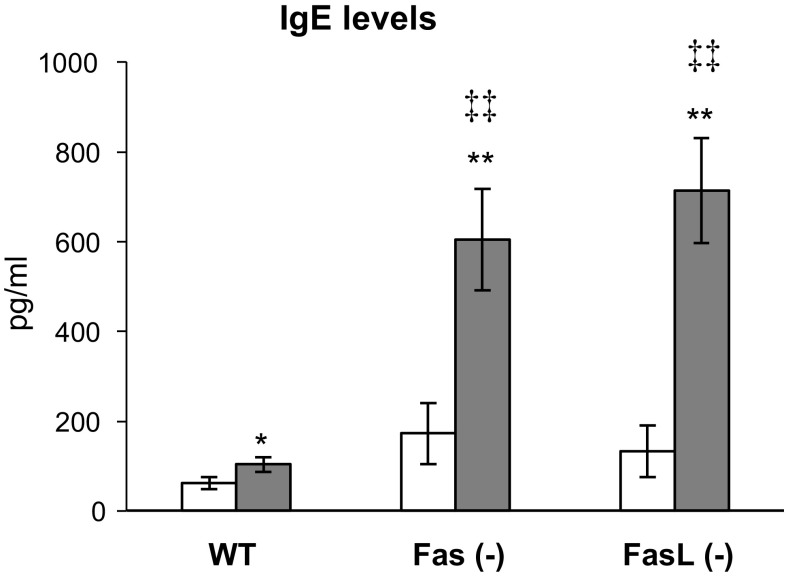
IgE antibody response in Fas (−), FasL (−) and WT (C57BL/6) mice sensitized with OVA or saline by the EC route. The* bars* represent the mean from 3 separate experiments ± SEM. *Significant differences with *p* ≤ 0.05, ** with *p* ≤ 0.01 in comparison to saline-treated mice, while ^‡‡^significant differences with *p* ≤ 0.001 in comparison to wild-type OVA-sensitized controls

To assess the influence of Fas/FasL pathway on specific IgE production sera were collected after 3rd. sensitization. As it can be seen from Fig. 6, specific IgE concentrations in OVA-sensitized mice were significantly increased in comparison to saline-sensitized mice of each strain (*p* ≤ 0.05). However, IgE concentrations in OVA-sensitized Fas- and FasL-deficient mice were higher than those observed in wild-type mice (*p* ≤ 0.01) (Fig. 6). This tendency was also observed in saline-sensitized Fas- and FasL-deficient mice when compared to wild-type (*p* ≤ 0.05) (Fig. 6).

### Cytokine expression in the skin

Here, we found that skin samples from OVA-sensitized Fas- and FasL-deficient mice showed significantly increased expression of mRNA for TGF-1β, IL-1β, IL-4, IL-5 and IL-13 in comparison to OVA-sensitized wild-type mice (*p* ≤ 0.05) (Table 2). On the other hand, levels of mRNA for CXCL9, CXCL10 and IL-17 were significantly decreased in OVA-sensitized skin samples from Fas- and FasL-deficient mice (*p* ≤ 0.05) (Table 2) in comparison to wild-type mice. We did not detect any mRNA for IL-10.

**Table 2:** Quantification of the mRNA for IL-1β, IL-4, IL-5, IL-10, IL-13, IL-17, TGF-1β, CXCL9 and CXCL10 in the OVA-sensitized skin sites in wild-type (WT), Fas (−) and FasL (−) mice

	WT	Fas (−)	FasL (−)
IL-1β	0.45 ± 0.01	125 ± 45**	198 ± 41**
IL-4	23.42 ± 2.09	719 ± 2.77	519.13 ± 12.12
IL-5	67.18 ± 7.46	433 ± 3.97	321 ± 2.12
IL-10	nd	nd	nd
IL-13	1.94 ± 0.55	40.5 ± 2.32	849 ± 2.34
IL-17	29 ± 0.99	3.1 ± 0.45**	4.05 ± 0.6**
TGF-1β	0.001 ± 0.0001	0.24 ± 0.005*	0.19 ± 0.008*
CXCL9	95.5 ± 13.58	5.67 ± 0.4*	6.45 ± 0.6*
CXCL10	114 ± 15.7	2.89 ± 0.6*	2.3 ± 0.78*

The data are expressed as the fold change in comparison to control (saline-treated skin samples). The results are mean ± SEM from three experiments

*nd* not detected

* Significant differences with *p* ≤ 0.05, while ** *p* ≤ 0.001 in comparison to wild-type mice. Number of mice in each group was 10

## Discussion

Here, we show that both apoptotic and non-apoptotic Fas signalling may play a role in AD pathogenesis by shaping the local dermal chemokine and cytokine microenvironment. Also, lack of Fas-induced apoptosis of a specific cell type at a specific time point of the local reaction may lead to further exacerbation of the local cytokine/chemokine milieu. Thus, Fas (but also other death receptors) may have broader function in the skin than previously suspected and may act as a potential “check-point” of further development of cutaneous inflammation.

Keratinocytes of healthy skin express the Fas receptor in low amounts [3]. In vitro studies using cells isolated from patients with AD have shown that T cells induce the expression of Fas on keratinocytes [3]. Fas ligand is either secreted from activated T cells or present on their surface and interacts with upregulated Fas on keratinocytes resulting in apoptosis [19]. Excessive keratinocyte apoptosis disrupts the integrity of the skin leading to altered barrier function, spongiosis and skin lesions, which favour invasion of allergens, and subsequent inflammation [2]. Balanced apoptosis of keratinocytes can therefore restrict skin inflammation to the region of contact with allergen, while prolonged survival of inflammatory cells contributes to the continuous immune response.

Apart from the first spontaneously occurring model of AD—the Nc/Nga mouse, a number of mouse models have been developed [20]. Spergel et al. (1998) have developed a mouse model of AD induced by repeated epicutaneous sensitization of tape stripped skin with ovalbumin. This model is characterized by increased scratching behaviour, the skin develops lesions characterized by epidermal and dermal thickening, infiltration of CD4+ T cells and eosinophils, there is also an increased deposition of collagen and upregulated expression of the Th2 cytokines IL-4, IL-5 and IL-13 [17]. By using this model in wild-type C57BL/6 mice, we found up-regulation of Fas expression not only on the cells infiltrating the dermis and epidermis but also on the keratinocytes localized at stratum spinosum, while FasL expression was detected only on the cells infiltrating the dermis and epidermis (Fig. 1). The mice also showed dermal thickening, fibrosis, infiltration of eosinophils and CD4+ T cells (Figs. 2, 3; Table 1). To further determine how Fas/FasL-apoptotic and non-apoptotic pathways are involved in the allergic skin inflammation, we used the epicutanous sensitization with ovalbumin in mice strains lacking Fas or FasL expression. Our results showed that lack of Fas or FasL expression actually leads to exacerbation of AD characteristics—the epidermal and dermal layers of Fas- or FasL-deficient skin show more thickening, fibrosis and inflammation in comparison to wild-type mice (Fig. 2). Of interest, mouse models of AD lack typical spongiosis formation [21] but in this study, we found signs of spongiosis in Fas- and FasL-deficient mice (Fig. 2a). This is in contrast with the paper by Trautmann et al. [3], claiming the role of Fas-induced keratinocytes apoptosis in spongiosis formation. Despite the lack of Fas or FasL expression, apoptotic cells were still detected in dermis and epidermis as infiltrating inflammatory cells or keratinocytes indicating involvement of other possible apoptotic pathways (Fig. 4). Fas and FasL- deficient mice showed actually increased numbers of apoptotic cells (Fig. 4). We have previously shown that lack of Fas or FasL expression in the HSV-2 infected epithelium leads to excessive inflammation, followed by increase of M30 + apoptotic cells identified as infiltrating inflammatory cells undergoing apoptosis through mitochondrial pathway [33]. Furthermore, there is an association between bfl-1 polymorphisms and the genetic predisposition to AD [22]. The anti-apoptotic bfl-1 gene is the only member of the Bcl-2 family that is transcriptionally regulated by inflammatory cytokines and might therefore be important in promoting the survival of effector T cells in patients with AD [22].

There are several ways to trigger apoptosis in keratinocytes: detachment of cells from tissue, triggering of the Fas receptor, activation of the perforin/granzyme B pathway, induction of genomic DNA damage and intracellular generation of ceramides catalysed by sphingomyelin hydrolase. Activated cytotoxic FasL+ T cells are able to kill Fas+ keratinocytes. Cytotoxic T cells and Th1-cells express both FasL and perforin, while Th2-cells express only FasL. Cytotoxic T cells mainly use the perforin pathway, while Th2-cells act through FasL and Th1-cells inconstantly use both possibilities [11]. Mice lacking Fas or FasL expression in this study showed no apoptosis of keratinocytes other than that related with infiltrating inflammatory cells, which indicates that Fas/FasL pathway indeed plays an important role in the keratinocyte removal from the atopic skin.

The inflammatory reaction within the sensitized skin of Fas- and FasL-deficient mice consisted mostly of macrophages, neutrophils and CD4+ T cells. This indicates that Fas-induced apoptosis plays an important role in controlling the magnitude and spread of the inflammation in AD. As mentioned above, Fas/FasL death receptors can also play a role in the inflammatory signalling [13]. Therefore, epidermal keratinocytes may act as enhancers of the inflammation through non-apoptotic Fas-triggered signalling and secretion of pro-inflammatory cytokines/chemokines that may attract further infiltration of different cell types. However, Fas-induced apoptosis of single cells in the epidermis may at the specific time point also act as a mechanism to remove cells with further signalling properties. Fas apoptosis resistance of epidermal keratinocytes may thus critically shape the epidermal outcome of infiltration in AD.

AD is characterized as a Th2 disease with the abundant production of Th2 cytokines, such as interleukin IL-4, IL-5 and IL-13 associated with eosinophilia and elevated serum IgE level. Animal models of AD have been showed to follow this characteristics [17, 21]. IL-4 and IL-13 have essential roles in the initial phase of tissue inflammation and are also responsible for the differentiation of allergen- specific Th2 cells and the class switching of activated B cells to IgE-producing cells. IL-5 also contributes to the increase and survival of eosinophils [19] and IL-13 takes part in fibrosis development [23]. In this study, we observed significantly increased expression of mRNA for IL-4, IL-5, IL-13 in the skin (Table 2) as well as increased serum IgE levels (Fig. 6) in OVA-sensitized wild-type mice. Furthermore, lack of Fas or FasL expression in OVA-treated mice led to significantly higher expression of mRNA for IL-4, IL-5 and IL-13 in comparison to wild-type mice. This reflected higher infiltration of CD4+ T cells and observed fibrosis (Table 1) in Fas- and FasL-deficient mice and indicates the role of Fas/FasL pathway in controlling the early phase of AD.

Additionally, we observed that lack of Fas/FasL-mediated apoptosis led to up-regulation of pro-inflammatory IL-1β. Interleukin 1 triggers adhesion molecule expression on endothelial cells, thereby contributing to the recruitment of phagocytic cells to the sites of infection [24]. Transgenic mice over-expressing the human caspase-1 precursor gene in epidermal keratinocytes (CASP1 transgenic mice) showed elevated serum levels of IgE and IgG1 at the age of 8 weeks, and mild pruritic dermatitis around the eyes and ears at the age of 16 weeks [25]. Histological examination showed prominent acanthosis, papillomatosis, hyperparakeratosis with dense infiltration of lymphocytes, neutrophils and mast cells, but not eosinophils in the skin lesion. In this study, increased expression of IL-1β is therefore directly related with the increased numbers of macrophages, neutrophils found in the skin samples of OVA-sensitized Fas- and FasL-deficient mice (Tables 1, 2).

CXC-type chemokines, including CXCL9 and CXCL10, are potent chemoattractants for activated T cells, NK cells, monocytes, dendritic cells and B cells [26]. We have previously demonstrated that lack of Fas or FasL expression disturbs mounting of the antiviral response during vaginal HSV-2 infection due to decreased production of CXCL9 and CXCL10 [27]. Here, we also observed disturbances in production of these two cytokines, which further confirms the role of Fas/FasL pathway in regulation of CXCL9 and CXCL10 production. However, we did not observe increased migration of CD8+ T cells in Fas- and FasL-deficient mice.

We may conclude that Fas/FasL-induced apoptosis plays an important role in controlling the development of Th2-driven inflammation characterized by dermal infiltration of CD4+ T cells and increased skin expression of Th2 cytokines.

Regulatory T cells (CD4+ CD25+, FoxP3+ Tregs) represent unique immune-regulatory cells in both humans and animals and play a central role in the maintenance of self-tolerance. By secreting IL-10 and/or TGF-β, different types of Tregs may directly or indirectly regulate differentiation, survival and activity of Th1, Th2, mast cells, eosinophils, keratinocytes and affect the isotype of antibodies synthesized by B cells [28]. Lack of Foxp3+ CD4+ CD25+ T cells leads to immune dysregulation and development of AD-like skin lesions, and enhanced Th2 responses. However, conflicting results regarding the numbers and functions of Tregs in AD have been reported. Some investigators demonstrated the absence of Foxp3+ Tregs in patient’s skin [29], while other authors [30] showed the elevated number of circulating CD4+ CD25+ Tregs with a normal suppressive function in patients with AD.

Several in vivo studies demonstrated that Treg numbers decline during an immune response as a result of Fas-dependent apoptosis [31, 32]. We previously showed that mice lacking Fas or FasL expression show disturbances in regulation of Treg numbers during immune response [33, 34]. Here, we found increased total numbers of circulating Tregs in OVA-sensitized Fas- and FasL-deficient mice but no differences in the draining lymph nodes (Fig. 4), which was further followed by increased production of TGF-1β, but not of IL-10 in the skin samples of Fas- and FasL-deficient mice (Table 2). We can therefore conclude that disturbances of Fas/FasL pathway may contribute to the maintenance of imbalanced Tregs numbers further influencing local inflammatory responses.

The IL-17-producing CD4+ T cells (Th17 cells) can be found in the epidermis and dermal infiltrate of AD thus playing a role in the pathogenesis of this disease [35]. Most notably, IL-17 is involved in inducing and mediating pro-inflammatory responses through up-regulation of GM-CSF, IL-8, TNF-α and the chemokine CXCL10 by keratinocytes and fibroblasts [36]. High levels of IL-17 were found in acute but not chronic lesions of AD suggesting that Th17 cells may act as an amplifier of the elicitation phase of AD [35]. Here, we found that expression of mRNA for IL-17 was decreased together with an expression of CXCL10 (Table 2), which further confirms the role of Fas/FasL signalling in development of the chemokine and cytokine microenvironment specific for AD.

In conclusion, our data show that loss of Fas activity strongly affects the early development of AD by leading to Th2-dominant inflammation characterized by dermal infiltration of CD4+ T cells, neutrophils and increased skin expression of Th2 cytokines. However, Fas/FasL-apoptotic pathway is also involved in restricting tissue remodelling and dermal fibrosis during AD.
